# Recommendation system in social networks with topical attention and probabilistic matrix factorization

**DOI:** 10.1371/journal.pone.0223967

**Published:** 2019-10-31

**Authors:** Weiwei Zhang, Fangai Liu, Daomeng Xu, Lu Jiang

**Affiliations:** School of Information Science and Engineering, Shandong Normal University, Jinan, China; Victoria University, AUSTRALIA

## Abstract

Collaborative filtering (CF) is a common recommendation mechanism that relies on user-item ratings. However, the intrinsic sparsity of user-item rating data can be problematic in many domains and settings, limiting the ability to generate accurate predictions and effective recommendations. At present, most algorithms use two-valued trust relationship of social network to improve recommendation quality but fail to take into account the difference of trust intensity of each friend and user’s comment information. To this end, the recommendation system within a social network adopts topical attention and probabilistic matrix factorization (STAPMF) is proposed. We combine the trust information in social networks and the topical information from review documents by proposing a novel algorithm combining probabilistic matrix factorization and attention-based recurrent neural networks to extract item underlying feature vectors, user’s personal potential feature vectors, and user’s social hidden feature vectors, which represent the features extracted from the user’s trusted network. Using real-world datasets, we show a significant improvement in recommendation performance comparing with the prevailing state-of-the-art algorithms for social network-based recommendation.

## Introduction

In daily life, with the continuous development of network information, information overload has become a serious challenge in an environment where users are overwhelmed, therefore, develop effective programs to help users locate information about their interests is coming to a creative and very important task that attracts the attention of research and application fields. For this purpose, recommended system (RS) are one of the important means of solving this problem. They help customers find what they are looking for and have been proven to drive sales and customer loyalty [1]. Collaborative filtering (CF) [2] is a common recommendation approach that has been adopted by many e-commerce sites, from Amazon [3] to Twitter [4] and YouTube [5], and is based on using neighbors to analyze the user’s interests by collecting the past behavior data of a large number of users, and identifying the neighbor users who have the same interests as the target user to forecast the interest or degree of the intended user for a certain event. Recently, collaborative filtering algorithms [6–8] tend to suffer from the serious problem of the natural sparsity of the user-item ranking data, which dues to each user only having rated a small segment of the available items.

To solve the problem of sparse data, various scholars have proposed effective solutions, including the introduction of auxiliary data, integrated cross-domain information or more hidden rules in mining data [9]. For example, item features have been utilized to improve the recommendation algorithm to deal with the problem of data sparsity. Considering a combination of data from different information sources to obtain cross-domain recommendation results, implicit feedback information in the data is mined making the most of information provided by the user or the combination of social relationship information, etc.

The recommendation algorithm combined with social information is an effective solution method to solve the disadvantage of data sparsity. In the social network, the direct trust network [10] can be extracted from each user’ friend lists, which can be available and is auxiliary data, solving the cold start disadvantage and enhancing the results of the recommendation. On one hand, in life, people usually consult with their friends before being making a decision and are affected by their acquaintances; for another, people also tend to be friends with similar people. Traditionally, we have only used the similarity of rating record calculations to measure the relationship between different users. Taking social networks into consideration, we can combine trust information and score records to prioritize the relationship between users to improve the quality of the recommendations. Although some previous studies have focused on auxiliary data, most of them use trust information directly and assume that if a user trusts his friend, he will like the same items as the people trusts [11–12]. In fact, users may not like these items, despite the fact that they are liked by people they trust, and therefore, the trusted person cannot influence target users in a certain probability.

More recently, some methods have taken text information data into account in addition to ratings [13–18]. Having investigated carefully, we have observed that the text information in a large proportion of recommended tasks can usually be divided into two categories: item specification [19–21]and user review [22–23]. An item specification called text information used to describe an item’s properties or attributes. For example, in an article recommendation such as a paper on cellulite, it refers to the title and summary of the paper. In Amazon recommendations and other item recommendations, the recommendation refers to item specifications and technical specifications. The other type is user reviews written by users, explaining why they favor or detest products according to their experiences. However, although both types of textual data have been found helpful to recommendation tasks, there are some intrinsic defects for them.

The matrix factorization technique [24] is one of the most widely used methods for solving the data sparsity and imbalance problems. Based on the latent factor model of matrix factorization, the commonly used matrix factorization methods mainly include normalized SVD, whose interaction information is mapped into space. Probability matrix decomposition (PMF) is a low-dimensional matrix approximate decomposition model, usually assume that the user’s interest is only affected by a few factors.

In summary, we comprehensively make full use of auxiliary information and propose an approach for a recommendation system in a social network with topical attention and probabilistic matrix factorization (STAPMF), which takes into account the influence of the trusted person and the target user’s own comments and predicts the user’s rating for items. The main dedications of framework are summarized as follows:

We introduce a novel recommendation model which is called STAPMF, which fuses attention-based recurrent neural networks to extract topical information from review documents and utilizes the trust information in social networks to learn feature vector.

We improved the MF model with social trust network and attention-based recurrent neural networks, which learn an adaptive factor that varies between users to weigh the impact of individuals and societies on decision-making. We believe that a definite person is not easily influenced by a trusted person, and vice versa.

The rest of the study is organized as follows. Section 2 reviews the related work. The model proposed in this study is presented in section 3. Section 4 shows the experiment results. Finally, the conclusion and future work are detailed in section 5.

## Related work

In this section, we will introduce the literature related to our work from the following three aspects.

### Probability matrix factorization

The motivation of the probability matrix factorization algorithm [25] is tantamount to adding the probability based on the matrix factorization model. Specifically, assuming that there are *M* users and an *N* item scoring matrix *R∈R*^*N×M*^, our goal is to reconstruct the scoring matrix by finding the potential model of the user and the product. In the collaborative filtering algorithm, the probabilistic matrix factorization model is used to study the eigenvectors of the users and the items, and then, the feature vectors are used to predict the score. We know that the conditional probability of the existing scoring data is:

$$p\left(R|U,V,\sigma_{R}^{2}\right)={\prod_{i=1}^{N}\prod_{j=1}^{M}\left[N\left(R_{\text{i},j}|g\left(U_{i}^{T}V_{j}\right),\sigma_{R}^{2}\right)\right]}^{I_{i,j}}$$ (1)

where *N(x*∣*μ*,*σ*_*R*_^*2*^*)* is the normal distribution with variance *σ*^*2*^ and mean *μ*, and *I*_*ij*_ is the indicator function that is equal to 1 if u has rated i; otherwise, it is equal to 0. The user’s actual score matrix *R* and the prediction score have a Gaussian distribution with a mean value of zero. Assume that the user eigenvector matrix *U∈R*^*N×M*^ and the commodity eigenvector matrix *U∈R*^*N×M*^ obey the Gaussian prior distribution with a mean of 0 and finally assume that the user score is independent of the same distribution, subject to spherical Gaussian prior distribution:

$$\text{p}\left(U|\sigma_{U}^{2}\right)=\prod_{i=1}^{N}N\left(U_{i}|0,\sigma_{U}^{2}I\right),\text{p}\left(V|\sigma_{V}^{2}\right)=\prod_{j=1}^{M}N\left(V_{j}|0,\sigma_{V}^{2}I\right)$$ (2)

Then, we use the Bayesian derivation of the user and the product of the implicit eigenvector posterior probability to perform the maximum likelihood estimate:

$$\begin{array}{l} \text{p}\left(U,V|R,\sigma_{R}^{2},\sigma_{U}^{2},\sigma_{V}^{2}\right)\propto \text{p}\left(R|U,V,\sigma_{R}^{2}\right)p\left(U|\sigma_{R}^{2}\right)p\left(V|\sigma_{V}^{2}\right)=\\ \prod_{i=1}^{N}\prod_{j=1}^{M}\left[N{\left(R_{i,j}|g\left(U_{i}^{T}V_{j}\right),\sigma_{R}^{2}\right)}^{I_{i.j}^{}}\right]\times \prod_{j=1}^{M}N\left(V_{j}|0,\sigma_{V}^{2}I\right)\times \prod_{i=1}^{N}N\left(U_{i}|0,\sigma_{U}^{2}I\right) \end{array}$$ (3)

**Fig 1:**
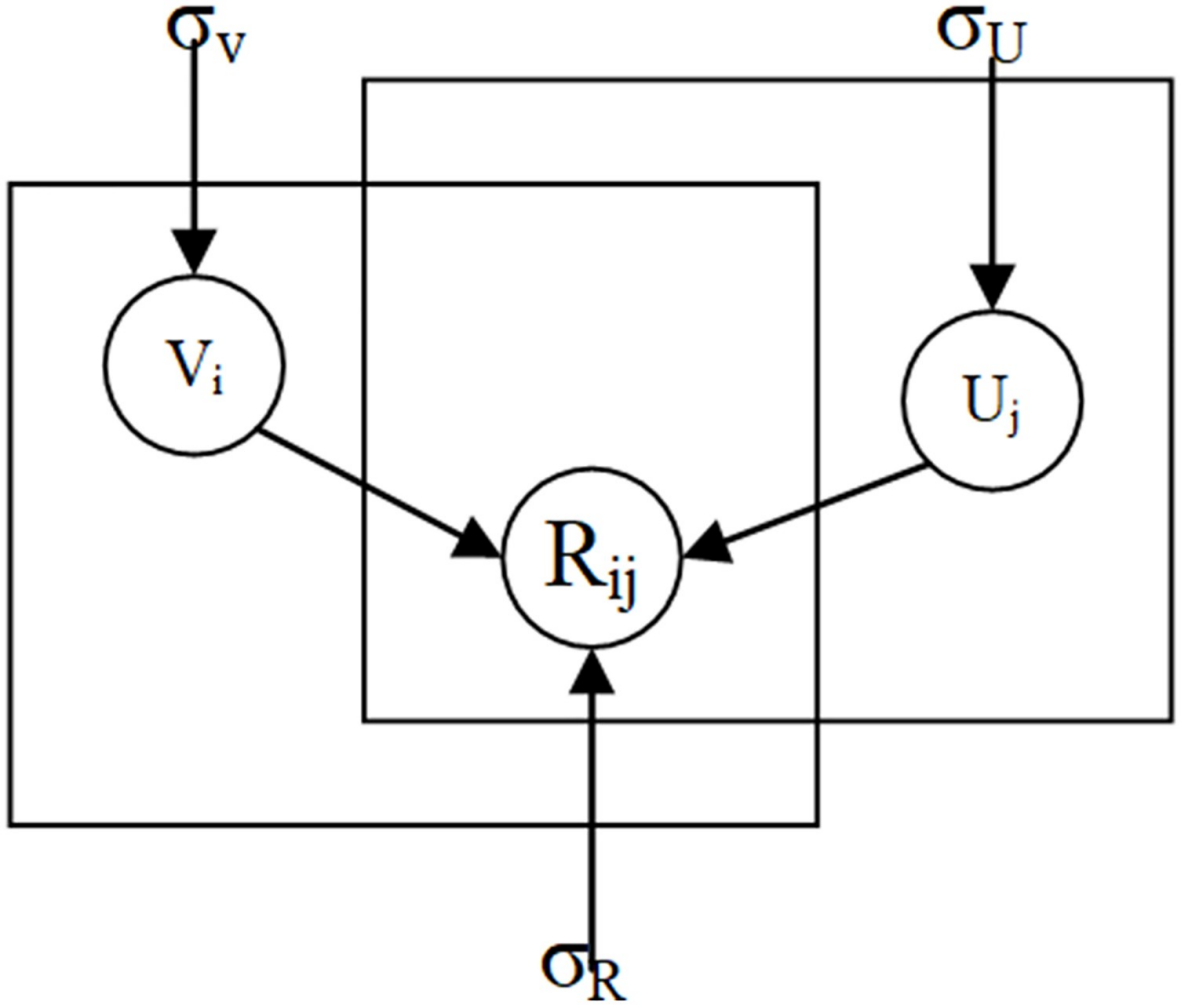
Probabilistic matrix factorization diagram model.

Fig 1 shows the graph of method. To prevent overfitting, the actual score is usually mapped to the [0, 1] interval by *f(x) = (x-1)/(R*_*max*_*-1)*, and the prediction score is mapped to the [0, 1] interval by *g(x) = 1/(1+exp(x))*. The final objective function is as follows:

$$L=\frac{1}{2}\sum_{i=1}^{N}\sum_{j=1}^{M}I_{i,j}{\left(R_{i,j}-g\left(U_{i}^{T}V_{j}\right)\right)}^{2}+\frac{\lambda_{U}}{2}\sum_{i=1}^{N}||U_{\text{i}}|{|}^{2}+\frac{\lambda_{V}}{2}\sum_{j=1}^{M}||V_{j}|{|}^{2}$$ (4)

### Deep neural networks in natural language processing

Recently, the application of NLP has gradually become another popular topic in the research of deep learning. In 2013, with the rise of word vector word2vec [26], many studies on the distributed feature correlation of various words emerged. At the beginning of 2014, researchers used different DNN models, such as convolution networks, cyclic networks and recursive networks, which made great progress in traditional NLP applications, including part of speech tagging, effective analysis, and syntactic analysis. [27] proposed employing an attention mechanism to achieve the most advanced results in machine translation [28] by using the neural language model.

The depth of learning in the research field of natural language processing tasks has been a great success, and thus, in the field of recommender systems, it has also attracted broad interest [29–30]. For example, [31] proposed to utilize convolution neural network embedding matrix factorization to learn features. Bahdanau et al [32] proposed an important concept of neurological attention. It is a weighted summation technique, but it can automatically analyze which parts are more important, such as words in an image or words in a sentence. With this attention mechanism, we can extract crucial words and sentences in the comment text and provide semantic explanations for the recommendations. Similarly, [33]utilized attention-based convolution neural networks to model review documents and obtained the most advanced results in the evaluation and prediction tasks. Wang et al. [34] proposed the DAMD model, using attention model to merge multiple prediction models for article recommendation.

### Social networks

The booming development of social network enables us to get user-generated content information from the Internet, such as social relations, tags, comments, etc. The recommended content also develops in a more diversified direction, including items, friends.

**Fig 2:**
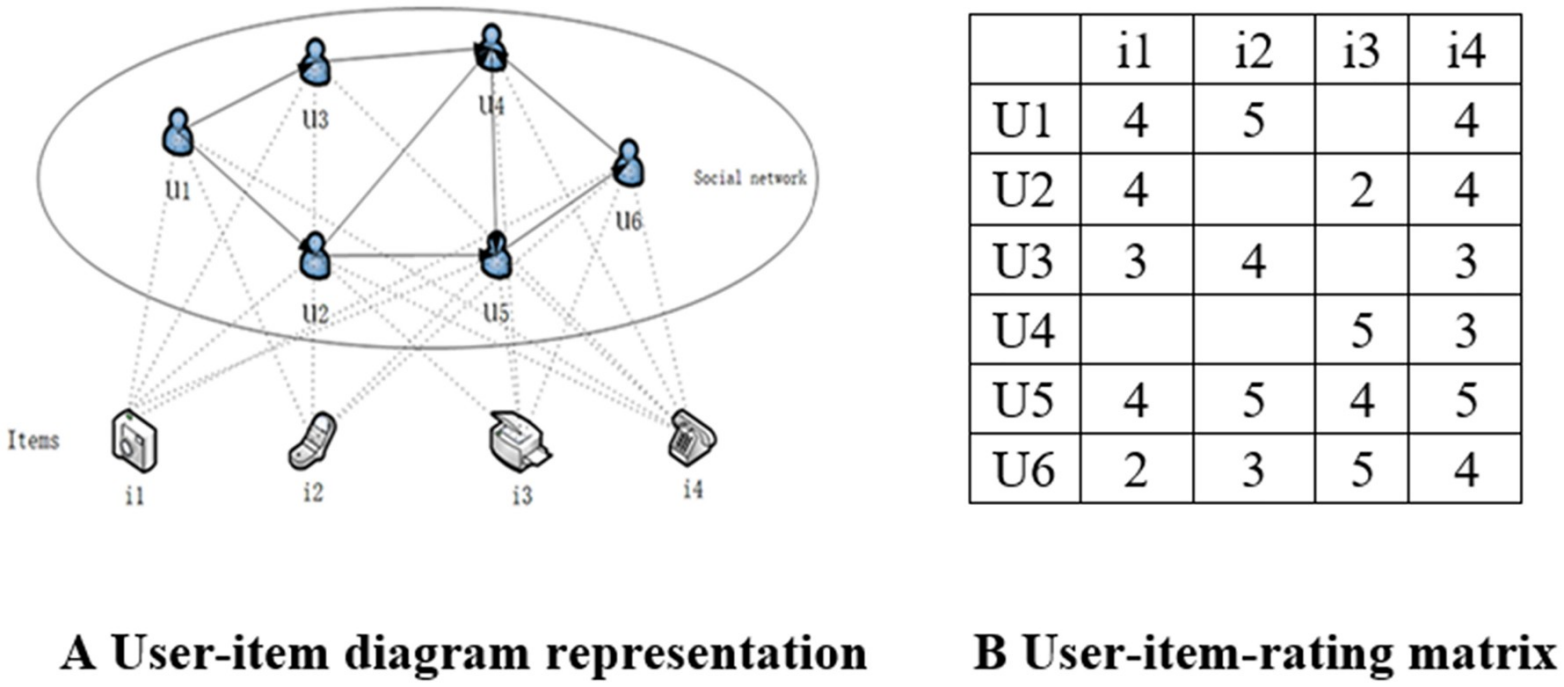
The expression of trust social network recommendations. A. User-item diagram representation. B. User-item-rating matrix.

Social network recommendation not only considers the relationship between users and items, but also adds social information between users, as shown in Fig 2A. The scoring prediction process integrates users’ scoring information of items shown in Fig 2B. and users’ trust, making the final scoring result more accurate.

## Proposed method

In this part, we will detail our proposed STAPMF model. We first describe our attention-based recurrent neural network architecture for document modeling, followed by a method for extracting text features from users and item review documents. We then introduce user social latent features from social networks, and we extend the traditional probability matrix probabilistic model by combining textual regularization. Finally, the parameters are optimized.

### Attention-based RNN for document modeling

We use a double-sided RNN with an attention mechanism to learn the features in the comment document. The model consists of four main components:(1) a word embedding layer (2) a sequence coding layer (3) a topical attention layer, and (4) a feature projection layer.

#### Word embedding layer

We initialize the word embedding layer with the pretrained word vector obtained from word2vec [35] and adjust it using backpropagation. The word embedding layer takes a series of words (*d*_*1*_, *d*_*2*_, *d*_*3*_ … *d*_*N*_) as input and maps each word to its respective k-dimensional vector representation *x*_*i*_*∈R*_*k*_.

#### Sequence encoding layer

Context annotations with the input sequence are supplied by the sequence encoding layer.[36–37] proposed the bidirectional gated recurrent unit (GRU) architecture, which is a popular variant of the normal recurrent hidden unit. By combining a forget gate with an input gate to synthesize a single update gate, and mixing cell and hidden states, each recurrent unit is capable of encapsulating sequential dependencies across different time scales.

Formally, a GRU computes its activation at time step *t* as the linear interpolation between the antecedently activation h_t-1_ and the candidate act ${\bar{\text{h}}}_{\text{t}}$.

$$h_{j}^{k}=(1-z_{j}^{t})h_{j}^{t-1}+z_{j}^{t}{\bar{h}}_{j}^{t}$$ (5)

$$net_{\text{z}}^{t}=x^{t}W^{z}+h^{t-1}Y^{z}$$ (6)

$$z_{j}^{t}=\sigma {\left(net_{z}^{t}\right)}^{j}$$ (7)

$$net_{\bar{h}}^{t}=x^{t}W+(r^{t}⊙h^{t-1})Y$$ (8)

$${\bar{h}}_{j}^{t}=\text{tanh}{\left(net_{\bar{h}}^{t}\right)}^{j}$$ (9)

$$net_{\text{r}}^{t}=x^{t}W^{r}+h^{t-1}Y^{r}$$ (10)

$$r_{j}^{t}=\sigma {\left(net_{r}^{t}\right)}^{j}$$ (11)

$$net_{y}^{t}=h^{t}W^{y}$$ (12)

$$y_{j}^{t}=\sigma {\left(net_{y}^{t}\right)}^{j}$$ (13)

where ⊙ is the Hadamard product, which is the product of the corresponding element; the subscript indicates the index of the node, and t indicates the time. *W*^*y*^*∈R*^*hd×yd*^ represents the parameter matrix of the hidden layer to the output layer and hd, yd is the amount of nodes in the hidden layer and the output layer, respectively. *W*^*z*^*∈R*^*xd×hd*^, *Y*^*z*^*∈R*^*hd×hd*^, respectively, represent the input matrix and the connection matrix of the hidden layer to the update gate z at the previous moment. *Xd* is the dimension of the input data. *W*^*r*^*∈R*^*xd×hd*^, *Y*^*r*^*∈R*^*hd×hd*^ are the input matrix and the connection matrix of the hidden layer to the reset gate r at the previous moment. *W∈R*^*xd×hd*^, *Y∈R*^*hd×hd*^ are the connection matrix of the input and the hidden layer to the selected state at the previous moment.

We want the annotations for each word to not only summarize the previous words but also summarize the words below. Hence, we employ a bidirectional GRU consisting of forward and backward GRUs. The activation functions of the forward GRU and the backward GRU at time step t are expressed by ${\vec{h}}_{j}^{t}$ and ${\overset{\leftarrow }{h}}_{j}^{t}$, respectively. At each time step, we connect forward activation and backward activation to obtain the final comment. That is ${\vec{h}}_{j}^{t}=\left[{\vec{h}}_{j}^{t},{\overset{\leftarrow }{h}}_{j}^{t}\right]$.

#### Topical attention layer

We assume that not all parts of the document are related to a particular topic. Therefore, we introduce attention mechanisms to capture the relative importance of different words. Suppose that each dimension of the reviews final representation vector is considered a topic related to the user’s characteristics or the items characteristics, and the value of each dimension represents the strength of the topic. For a specific topic, each word in the review contributes differently to the topic, so we propose an attention weighting method, that is, each word learns an attention for each topic, and then the representation vector of the topic is weighted.

Consider, for example, the k-th attention module. Given the sequence of word annotations (*h*_*1*_, *h*_*2*_, *h*_*3*_ … *h*_*n*_), the attention module first transforms each word annotation through a single layer perceptron with the tanh activation function:

$$q_{t}^{k}=\text{tanh}(W_{s}^{k}h_{j}^{t}+b_{s}^{k})$$ (14)

Then, the attention module compares the similarity between the context vector zk and the transformed annotation by calculating the dot product and assigns a weighted score to each annotation using the softmax function:

$$a_{t}^{k}=\frac{z_{k}\cdot q_{t}^{k}}{\sum_{t=1}^{n}z_{k}\cdot q_{t}^{k}}$$ (15)

Then, the attention module weights the representative vector of the topic:

$$\bar{h_{k}}=\sum_{t=1}^{n}a_{t}^{k}h_{t}$$ (16)

Feature Projection Layer. After the representative vector of the topic is obtained, the projection layer obtains a value indicating the topic in the vector.

$$d_{k}=\text{tanh}(W_{c}^{k}{\bar{h}}_{k}+b_{c}^{k})$$ (17)

The representation vector of review is *d = [d1*, *d2 …dk]*, the representation vector of user reviews and item reviews is the average of the review representation vectors.

### Extracting textual features from review documents

First, we define the comment text of user i as *d*_*u*,*i*_, which is all of user’s comments. Similarly, the relevant comment text of item j is *d*_*v*,*j*_. The recursive neural network based on user comment document and item comment document is called user attention network and item attention network respectively. Considering the review document, we first use an attention-based recurrent neural network to generate potential document representations for each individual comment and then average them as text features extracted from the review document:

$$U_{i}=UAN(W,X)+\varepsilon_{i},\varepsilon_{i}∼N\left(0,\sigma_{u}{}^{2}I\right)$$ (18)

$$V_{j}=IAN(Z,Y_{j})+\varepsilon_{j},\varepsilon_{j}∼N\left(0,\sigma_{v}{}^{2}I\right)$$ (19)

where *UAN* refers to the user attention network, the function denotes the textual features for user i generated by feeding the user review document *D*_*u*,*i*_ into the user attention network with parameters *W*. where the function denotes the textual features for item j generated by feeding the item review document D_v,j_ into the item attention network with parameters *Z*. *IAN* refers to the item attention network.

We suppose that the user and the item underlying factors are highly correlated with text features extracted from the review document. Therefore, we can get the prior probability distribution of users and the potential factor vector of the items.

$$p(U|W,X,\sigma_{U}^{2})=\prod_{\text{i}}^{N}N\left(U_{i}|UAN(W_{},X),\sigma_{U}^{2}I\right)$$ (20)

$$p(V|Z,Y,\sigma_{V}^{2})=\prod_{\text{j}}^{M}N\left(V_{j}|IAN(Z,Y_{j}),\sigma_{V}^{2}I\right)$$ (21)

where U_i_ and V_j_ are the textual features extracted from the comment texts of user i and item j respectively.

### User social latent feature

For the extraction of user characteristic vectors, this part takes the adjacency matrix called the trust matrix *T* = [*T*_*il*_]_*n×m*_. From the perspective of reality, there exists an asymmetric matrix S, because the user relationship network is oriented, and the target user trusts the user does not mean that the user will trust the target user. For each pair of users, the trust value is 1, otherwise 0. The individual potential characteristics of user I are represented by *Ui*, while the social potential characteristics are represented by *Si∈Rm×l*. The potential feature vectors of each society obey the gaussian prior distribution with a mean value of 0, and the specific form is shown as follows

$$p(S|\sigma_{s}^{2})=\prod_{i=1}^{N}N(S_{i}|0,\sigma_{S}^{2}I)$$ (22)

By directly trusting the feature vector, the average feature vector *Ui* of target user i can be obtained. It can be represented as:

$$U=\frac{\sum_{l=1}^{n}T_{il}U_{l}}{\sum_{l=1}^{n}T_{il}}$$ (23)

At the same time, the trust matrix is normalized so that *∑*^*n*^_*l=1*_*T*_*il*_ = 1. Now, we can rewrite the formula as:

$${\bar{U}}_{\text{i}}=\sum_{l=1}^{n}T_{il}U_{l}$$ (24)

As is known, we can be influenced by trusted people, but not be exactly like them, but the characteristics of the people we trust are not completely transferred to the target users. So, *T* and *U* are not the same. Now, we calculate the conditional distribution of *T* given by the potential characteristics of the individual and the trust relationship as follows:

$$\begin{array}{l} p\left(S|U,T,\sigma_{s}^{2},\sigma_{T}^{2}\right)\\ \propto P\left(U,T|S,\sigma_{T}^{2}\right)\times P\left(S|\sigma_{S}^{2}\right)\\ =\prod_{\text{i}=1}^{N}\prod_{j=1}^{M}N(\sum_{l=1}^{N}S_{\text{il}}U_{l}|S_{i},\sigma_{T}^{2}I)\times \prod_{i=1}^{N}N(S_{i}|0,\sigma_{S}^{2}I) \end{array}$$ (25)

Now, we have an item with two different prediction scores based on individual characteristics and potential social characteristics of the target users. One needs to consider only the personal characteristics of the users regardless of the user’s features users from trusted out of the man to predict. In real life, some people decide not to consider the opinions of people they trust when choosing an item. Even a trusted person can’t easily change his decision. Others are easily influenced by trusted people and their opinions are easily changed. Therefore, the same user is affected by the potential characteristics of different societies, and only from the perspective of user trust is not comprehensive enough.

**Fig 3:**
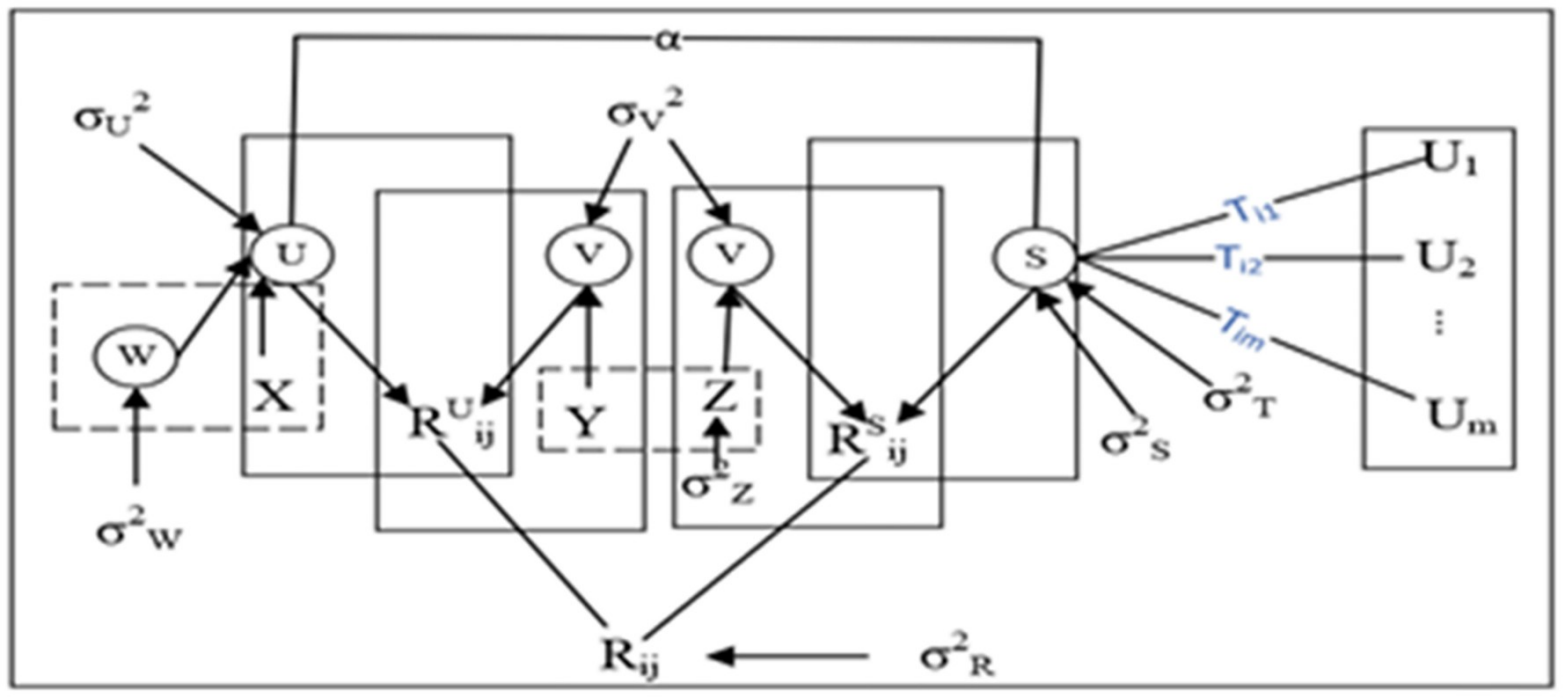
STAPMF model.

Adaptive weighting of each user’s personal and social underlying characteristics. As shown in Fig 3. *α∈ R*_*m×k*_ is the influence factor, and 1-*α*_*i*_ is the influence degree of users. And assume that it obeys the gaussian prior distribution of one half mean

P(α|σα2)=∏i=1NN(αi|12,σα2) (26)

### Optimization methodology

The user and the item potential factor vector *U*, the user social potential feature S, the item potential factor vector *V* and the firm factor *α* are synthesized to make better recommendations on the social network. According to the formulas (25) and (26), then, through Bayesian reasoning, the formula for the posterior distribution of the potential factors of the user and the product is given as follows:

P(U,S,V,Z,W,α|R,X,Y,σR2,σU2,σS2,σV2,σα2,σz2,σw2)∝P(R|U,S,V,α,σR2)×P(S|U,S,σS2)×P(U|W,X,σU2)×P(V|Z,Y,σV2)×P(S|σS2)×P(W|σW2)×P(Z|σZ2)P(α|σα2)=12∏i=1N∏j=1M[Iij(Rij−g(αiUi+(1−αi)S)iVjT]2×∏i=1NN(∑i=1NSikUk|Si,σs2I)×∏i=1NN(Ui|UAN(W,Xi,σU2I))×∏i=1NN(Si|0,σs2I)×∏j=1MN(Vj|IAN(Z,Yj,σV2I))×∏i=1NN(αi|12,σα2) (27)

Keep the super parameter *σ*^*2*^, *σ*^*2*^_*U*_, *σ*^*2*^_*V*_, *σ*^*2*^_*W*_ constant and maximize formula (27) to minimize the squared function:

$$\begin{array}{l} L=\frac{1}{2}\sum_{i=1}^{N}\sum_{j=1}^{M}I_{i,j}{\left(R_{i,j}-g(\alpha_{i}U_{i}+(1-\alpha_{i})S_{i})V_{j}^{T}\right)}^{2}\\ +\frac{1}{2}\lambda_{T}\sum_{i=1}^{N}\left((\sum_{l=1}^{n}T_{ik}U_{k}-S_{i})(\sum_{l=1}^{n}T_{ik}U_{k}-S_{i}{)}^{T}\right)\\ +\frac{1}{2}\lambda_{U}\sum_{i=1}^{N}{\left\|U_{i}-UAN\left(W,X_{i},\sigma_{U}^{2}I\right)\right\|}_{F}^{2}+\frac{\lambda_{U}}{2}\sum_{i=l}^{N}S_{i}S^{T}\\ +\frac{\lambda_{V}}{2}\sum_{j=1}^{M}{\left\|V_{j}-IAN\left(Z,Y_{j},\sigma_{V}^{2}I\right)\right\|}_{F}^{2}+\frac{\lambda_{\alpha }}{2}\sum_{i=1}^{N}\alpha_{i}^{2}+\frac{\lambda_{W}}{2}\sum_{a=1}^{\left|W_{a}\right|}{\left\|W_{a}\right\|}^{2}+\frac{\lambda_{Z}}{2}\sum_{b=1}^{\left|Z_{b}\right|}{\left\|Z_{b}\right\|}^{2} \end{array}$$ (28)

where *λ*_*U*_ = *σ*^*2*^*/σ*^*2*^_*U*_, *λ*_*V*_ = *σ*^*2*^*/σ*^*2*^_*V*_, *λ*_*S*_ = *σ*^*2*^*/σ*^*2*^_*S*_, *λ*_*α*_ = *σ*^*2*^*/σ*^*2*^_*α*_ and ‖●‖_F_ denote the Frobenius norm, and the method of gradient descent is adopted to estimate parameters, and the formula is as follows:

$$\begin{array}{l} \frac{\partial L}{\partial U_{i}}=-\sum_{j=1}^{M}\alpha_{i}I_{ij}^{R}(R_{i,j}-g\left(\alpha_{i}U_{i}+(1-\alpha_{i})S_{i}\right){\overset{}{V}}_{j}^{T})\\ \times g^{\prime }(\left(\alpha_{i}U_{i}+(1-\alpha_{i})S_{i}\right){\overset{}{V}}_{j}^{T}){\overset{}{V}}_{j}+\lambda_{T}\sum_{k=1}^{n}S_{ik}+\lambda_{u}U_{i} \end{array}$$ (29)

$$\begin{array}{l} \frac{\partial L}{\partial S_{i}}=-\sum_{j=1}^{M}\left(1-\alpha_{i}\right)I_{ij}^{R}(R_{i,j}-g\left(\alpha_{i}U_{i}+(1-\alpha_{i})S_{i}\right){\overset{}{V}}_{j}^{T})\\ \times g^{\prime }(\left(\alpha_{i}U_{i}+(1-\alpha_{i})S_{i}\right){\overset{}{V}}_{j}^{T}){\overset{}{V}}_{j}+\lambda_{S}\sum_{k=1}^{n}(S_{ik}-S_{k})+\lambda_{s}S_{i} \end{array}$$ (30)

$$\begin{array}{l} \frac{\partial L}{\partial V_{j}}=-\sum_{i=1}^{N}I_{ij}^{R}(R_{i,j}-g\left(\alpha_{i}U_{i}+(1-\alpha_{i})S_{i}\right){\overset{}{V}}_{j}^{T})\\ \times g^{\prime }\left(\left(\alpha_{i}{\overset{}{U}}_{i}+(1-\alpha_{i})S_{i}\right){\overset{}{V}}_{j}^{T}\right)\left(\alpha_{i}U_{\text{i}}+\left(1-\alpha_{i}\right)S_{i}\right)+\lambda_{v}V_{j} \end{array}$$ (31)

$$\begin{array}{l} \frac{\partial L}{\partial \alpha_{i}}=-\sum_{i=1}^{M}I_{ij}^{R}(R_{i,j}-g\left(\alpha_{i}U_{i}+(1-\alpha_{i})S_{i}\right){\overset{}{V}}_{j}^{T})\\ \times g^{\prime }\left(\left(\alpha_{i}{\overset{}{U}}_{i}+(1-\alpha_{i})S_{i}\right){\overset{}{V}}_{j}^{T}\right)\left(U_{i}-S_{i}\right){\overset{}{V}}_{j}^{T}+\lambda_{\alpha }\alpha_{i} \end{array}$$ (32)

When *U* and *V* are assumed to be constant, the goal of the network for users and products is to adjust its internal weights W and Z to make the extracted text features close to the target’s *U* and *V*.

$$L_{W}\left(X_{i},U_{i}\right)={\left\|U_{i}-UAN\left(W,X_{i},\sigma_{U}^{2}I\right)\right\|}_{F}^{2}$$ (33)

$$L_{Z}\left(Y_{j},Z\right)={\left\|V_{j}-IAN\left(Z,Y_{j},\sigma_{V}^{2}I\right)\right\|}_{F}^{2}$$ (34)

While *U* and *V* are fitted with alternating least squares, *W*_*U*_ and *W*_*V*_ are optimized with mini-batch gradient descent.

$$W≔W-\eta \nabla E_{P}\left(W\right),Z:=Z-\eta \nabla E_{P}\left(Z\right)$$ (35)

In the optimization process, *U*, *V*, *T*,*α*, *W*_*U*_, and *W*_*V*_, are alternately updated until the results converge, and the user can predict the unknown score of the project through the optimization process.

$${\text{R}}_{\text{i},\text{j}}\approx \text{E}\left[{\text{R}}_{\text{i},\text{j}}|\text{g}\left(\alpha_{\text{i}}{\text{U}}_{\text{i}}+\left(1-\alpha_{\text{i}}\right){\text{S}}_{\text{i}}\right){\text{V}}_{\text{j}}^{\text{T}},\sigma^{2}\right]$$ (36)

## Experiment

### Datasets and evaluation protocol

To verify the validity of our model in rating prediction, we used two real Epinions and Ciao data sets, which contain both commentary and online social data collected and published by Tang et al.[38] in 2011. Epinions and Ciao are two well-known mass consumer reviews websites with major markets in the US and Europe. On both sites, any user can rate and comment on all items after signing up, as well as browse other users’ ratings and reviews to help them make more favorable decisions. At the same time, users can establish a friendship relationship with trusted users to establish a social network. First of all, we randomly selected 80% of the data from the data set as training data, and the rest data were evenly divided into two parts, namely verification data and test data. The size of used datasets is presented in Table 1.

**Table 1:** Data statistics on two real-world datasets.

Datasets	#users	#items	#ratings	#trust
Epinions	22166	296277	922267	355754
Ciao	7375	106797	284086	111781

The evaluation index in the experiment is the root mean square error (RMSE). The smaller RMSE value, the better performance of the recommended algorithm. RMSE is defined as

$$RMSE=\sqrt{\frac{\sum_{i,j}^{N,M}{\left(R_{i,j}-{\hat{R}}_{i,j}\right)}^{2}}{R_{C}}}$$ (37)

where *R*_*C*_ is the test dataset, *R*_*i*,*j*_ is the real value, and *ʌR*_*i*,*j*_ is the predicted value.

In order to evaluate the recommendation accuracy of our algorithm, we compared the following algorithms.

The probability matrix factorization (PMF) [30] is a classical rating prediction model, but it uses rating information in the collaborative filtering recommendation, ignoring the impact of the relationship between the users and the text information.

The SoRec[39] algorithm adopts matrix decomposition method and assumes that recommendation system and social network share the same user implicit space. User implicit space is a matrix composed of multiple vectors, each of which is the user’s implicit eigenvector

SoReg[40] was proposed by Ma et al. and uses the similarity of ratings as a measure of trust between users, and the main mode of communication in SocialMF is improved.

RSTE[41] proposes a matrix decomposition framework with social regularization and explains the differences between social recommendation system and trust perception recommendation system

TrustMF[42] was proposed Yao et al. to separately model the situation when the user is a trustee and a trusted person and integrates the users to obtain the recommended result.

Recommendation based on social relations (SocialMF)[43] proposed integrating the user’s social relations into the recommendation system, but this model project feature vector does not take into account the additional information of the item product.

Collaborative Topic Regression (CTR) [44] learns interpretable latent structures from user-generated content so that probabilistic topic modeling can be integrated into collaborative filtering.

Social relationships based on marginalized stacked Denoising Autoencoders(SDAE) [45] proposed a deep learning method to learn user preferences and the social influence of friends simultaneously when generating recommendations.

### Experimental results and analysis

A set of experiments considered the influence of the characteristic dimension of experimental results; for the other parameters, we adjust the parameters in advance for each method, and the optimum value is used for all experiments. The table indicates that the potential feature dimension *K* of all algorithms is the RMSE value under different value conditions, and the dimension of the hidden feature vector *K* is set to 5, 10, and 20, the results are shown in Table 2.

**Table 2:** RMSE comparisons for different K.

	K = 5		K = 10		K = 20	
Algorithm	Epinions	Ciao	Epinions	Ciao	Epinions	Ciao
PMF	1.1347	1.0483	1.1491	1.0438	1.1581	1.0455
SoRec	1.0758	1.0308	1.1047	1.0645	1.2260	1.1620
SoReg	1.0674	0.9924	1.1109	1.0011	1.2399	1.0970
RSTE	1.0915	1.0471	1.1387	1.0300	1.2291	1.1656
SocialMF	1.0809	1.0087	1.0904	1.0700	1.1932	1.0060
TrustMF	1.0722	0.9794	1.1208	0.9823	1.2893	0.9812
CTR	1.0644	0.9543	1.0576	0.9662	1.0916	0.9678
SDAE	1.0457	0.9321	1.0501	0.9525	1.0732	0.9603
STAPMF	1.0301	0.9131	1.0416	0.9341	1.0478	0.9517

The following conclusions are obtained by comparison: Our method STAPMF performs best in all cases. The social recommendation algorithms (such as SocialMF and SoReg) are superior to RMSE in the probability matrix factorization method PMF, which only relies on the user rating. This shows that making full use of the implicit user preference information in social relationships can effectively improve the precision of the recommendation algorithm. Collaborative topic regression learns interpretable potential feature vectors from user-generated content, which also suggests that leveraging user reviews and product information can help to improve recommendation algorithms. The algorithm shows good recommendation ability in Epinions and Ciao data sets, and verifies the reliability of this algorithm, and has no obvious error for specific data sets.

In this paper, STAPMF has a certain degree of performance degradation when the dimension is increased, but it still has obvious competitive advantages compared with other algorithms. Since the algorithm in the paper models each user from two dimensions, including (rating, truster), the dimension of each user is actually twice that of a single vector dimension, which enhances the fitting ability of the algorithm. It also increases the risk of overfitting. Table 1 also shows that the performance at dimension 5 is better than the other dimensions. Therefore, this algorithm is more suitable for adopting a lower vector dimension.

We will explore the different settings of hyperparameters that affect the performance of our proposed model. The hyperparameters in the experiment include the dimension d_W_, d_Z_ of word embedding, the state dimension of the sequence encoder d_X_, d_Y_. the dimension in the attention mechanism module d_A_. On the Ciao data set, *λ*_*u*_ is set to 0.1, 1, 10, 30, 40, 100, respectively, and *λ*_*v*_ is set to 1, 10, 30 respectively. The parameters *λ*_*u*_ set on the Epinions data set are 0.01, 0.1, 1, 10 respectively, *λ*_*v*_ are 10, 30, 100 respectively.

**Fig 4:**
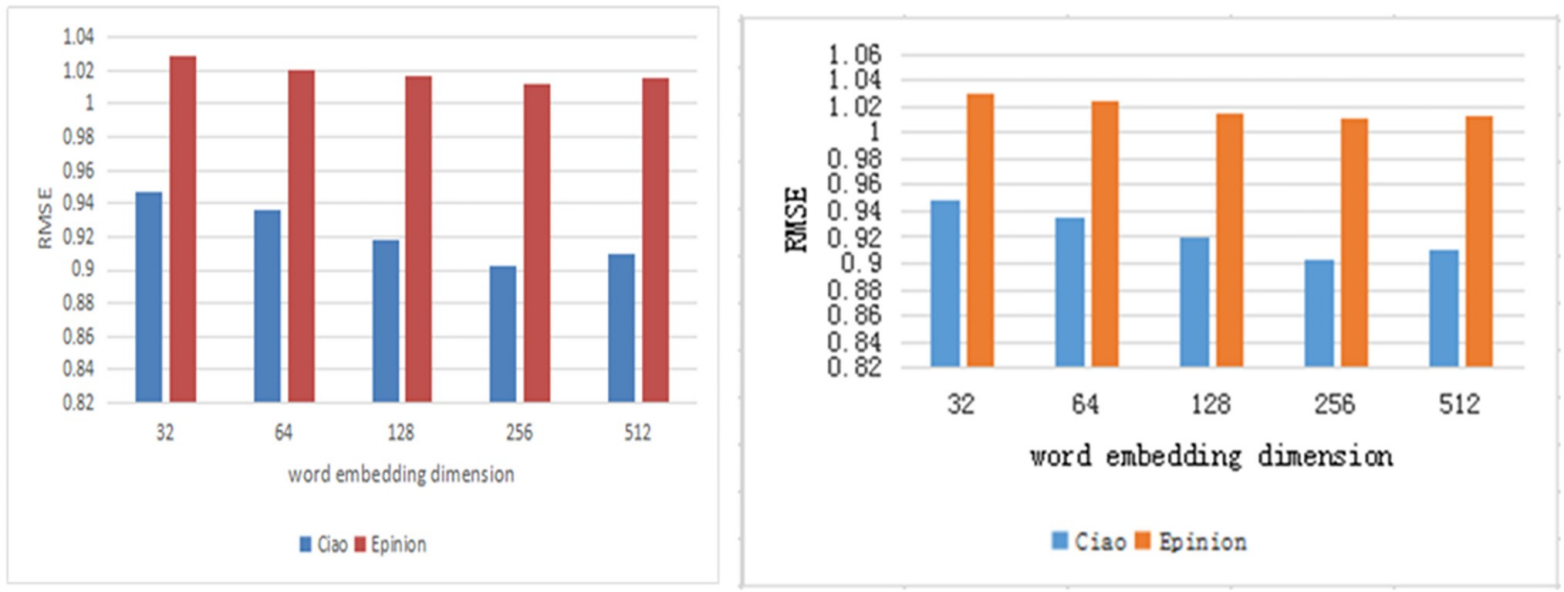
Validation RMSE as a result of varying d_w_, d_z_.

**Fig 5:**
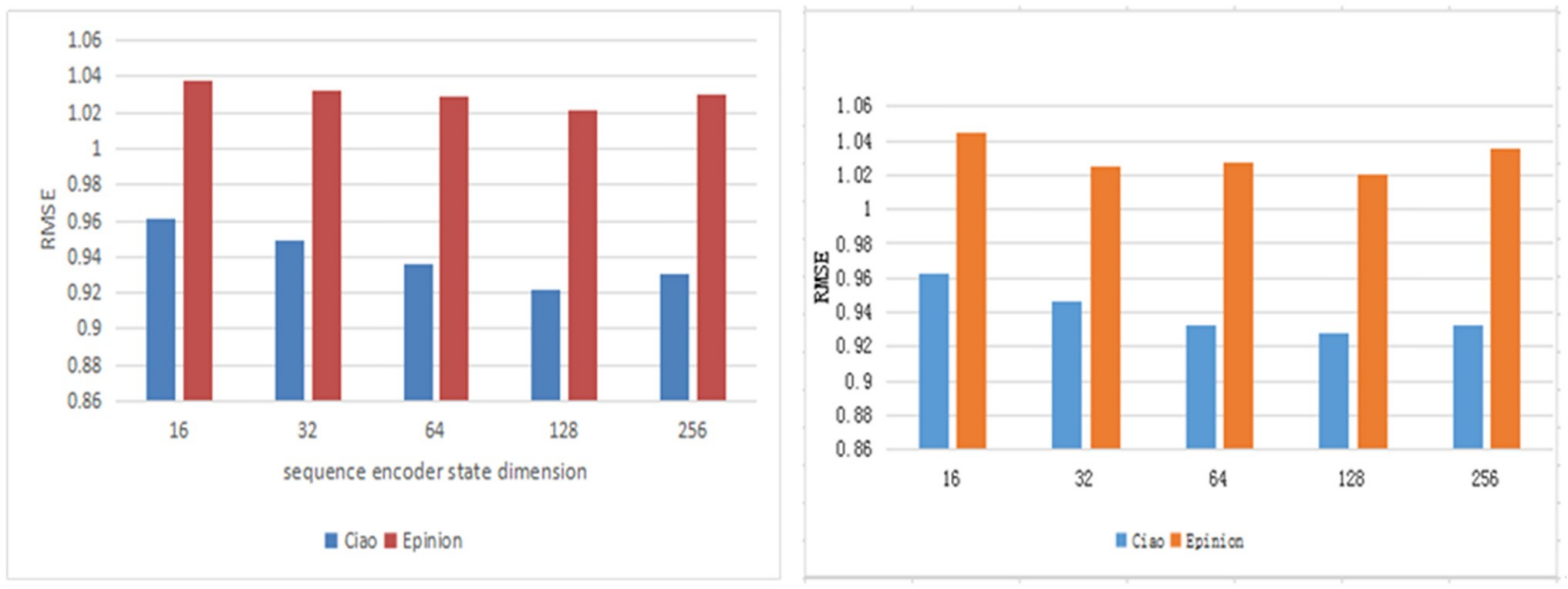
Validation RMSE as a result of varying d_x_, d_y_.

**Fig 6:**
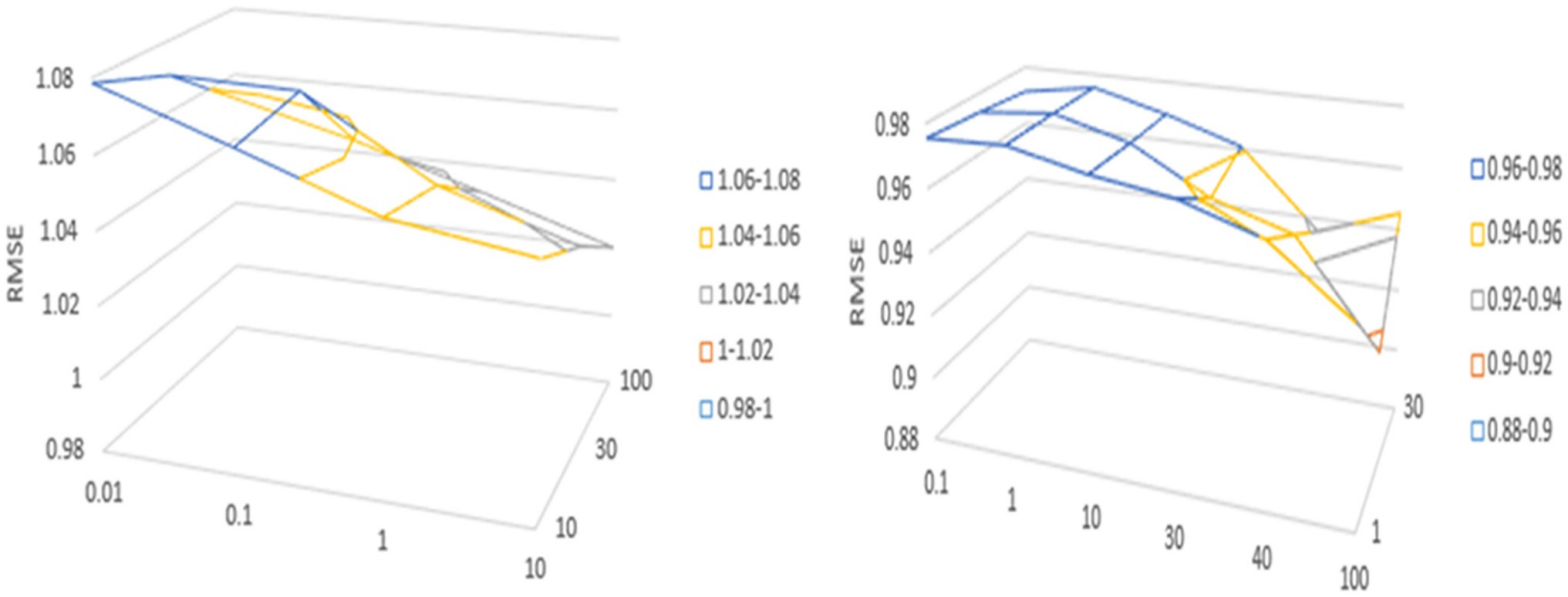
Validation RMSE as a result of varying λ_u_, λ_v_.

According to the experimental results, as shown in Figs 4 and 5, we set the embedding dimension to 256, the sequence encoder state dimension to 128, and the dimension of the conversion annotation in the attention mechanism module to 128. We observe *λ*_*u*_, *λ*_*v*_. The effect of the recommendation is significant, and the effect is shown in Fig 6. In these two data sets, when *λ*_*u*_ and *λ*_*v*_ take the value of 100, the effect is the best. When the value of *λ*_*u*_ increases continuously, the user vector is not updated. The user implicit feature is projected into the project implicit feature vector space mainly through Describe the characteristics obtained by the file.

**Fig 7:**
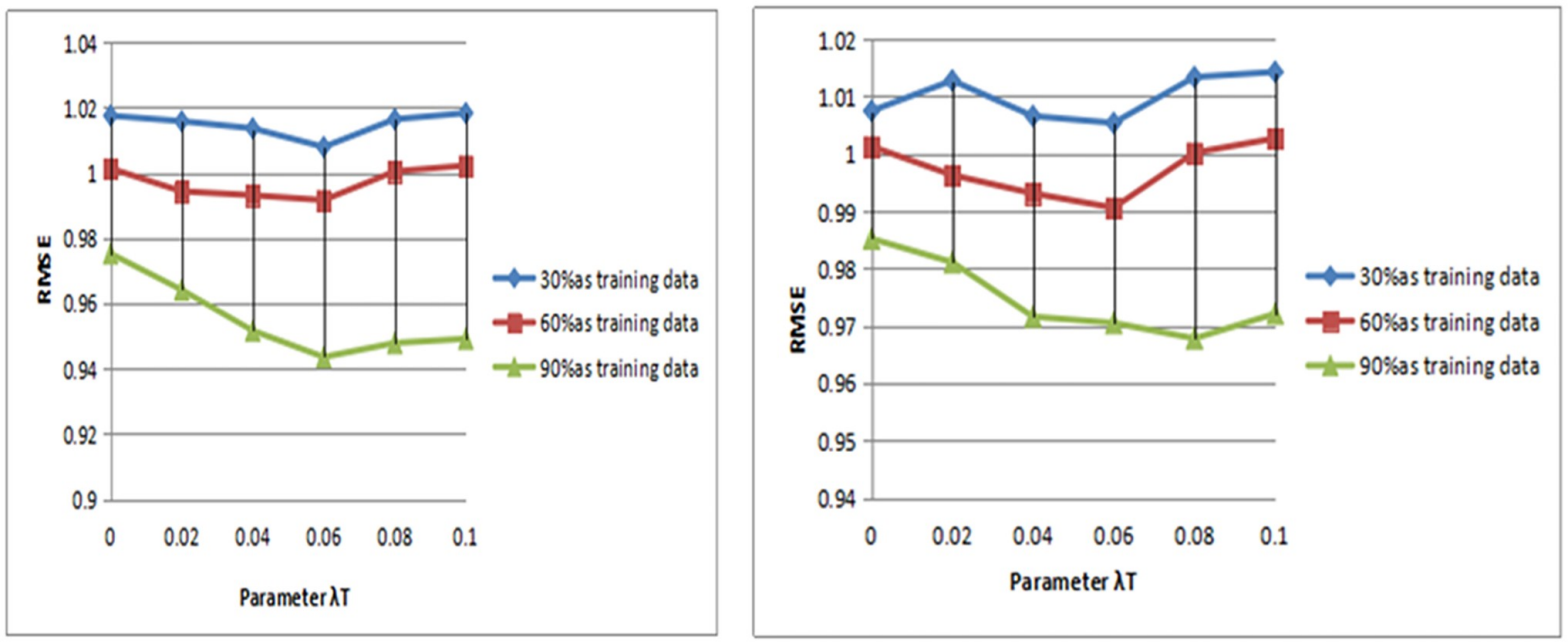
Parameter analysis of Ciao.

**Fig 8:**
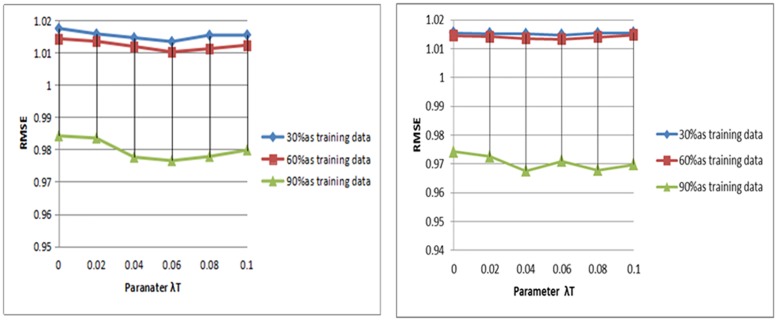
Parameter analysis of Epinions.

In our STAPMF approach, the parameter *λ*_*T*_ is the degree to which the trusted regular term influences the objective function (25). However, the maximum value of the value will lead to the social trust information to dominate the prediction model, which may limit the accuracy of the prediction. In this part, we use different sizes of training data and analyze the influence of different parameter values on the algorithm results. The influence of *λ*_*T*_ value between 0 and 1 on prediction accuracy is studied. For the other parameters, we use the grid search method to figure out the best combination of values. And the default is k = 5 and k = 10, and λu = λv = 0.1. Figs 7 and 8 compare the RMSE values of the model are different. As presented in the results, as the RMSE value increases, the RMSE value begins to decrease. When a threshold value is exceeded, the RMSE value increases again.

**Fig 9:**
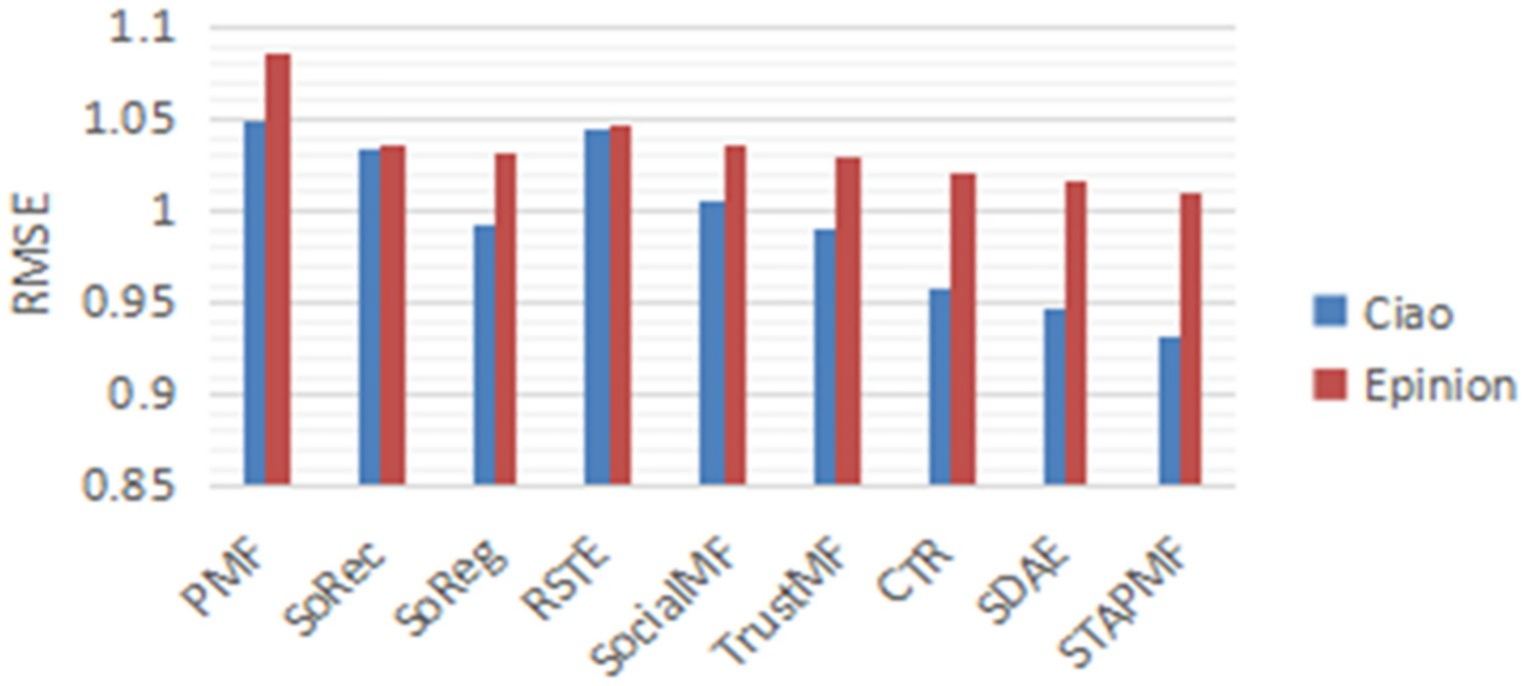
Comparison results on the different datasets.

Another challenge in the recommendation system is cold start. In order to verify the efficiency of our method in cold start, we conducted experiments on two data sets and compared them with other methods. Fig 9 shows the RMSE results for the three datasets used only for cold-start users. It can be seen that compared with other algorithms, the proposed STAPMF algorithm can provide the most precise rating forecast for cold start users. The main reason is not only considering the influence of trust network, but also the individual features of each user are adaptive to balance item and social features.

## Conclusions

Although the recommended algorithm achieved great success in a large number of practical applications, the recommendation system is still subject to issues such as data sparsity, imbalance and cold start, and the user’s decision on the Internet item is influenced by its own characteristics and the recommendation of a trusted friend. A local attention probability matrix factorization (STAPMF) method based on social network is proposed. Our STAPMF method includes three stages of learning. Firstly, we exploit a topical attention model to learn the latent feature vectors of users and items. Secondly, we take into consideration the influence of the community impact in the trusted social network. Finally, the proposed objective function is minimized with the users’ characteristics and the user social latent feature vectors. Unlike the most existing models, our model leverages the user review information and the impact of the trust networks and sets the impact factors. Our experimental analysis of the two datasets, Ciao and Epinions, shows that our method outperforms traditional recommendation algorithms and recommendation algorithms based on social networks. In addition, the proposed algorithm performs better than the existing algorithms in handling cold start.

The trust relationship of users in the social trust network is constant while the trust relationship can change with time. In addition, ratings and comments from users are also time sensitive, and outdated comments may become noise information for recommendations. Therefore, the time sensitivity can be incorporated into the trust social network in future works.

## Supporting information

S1 File The proposed STAPMF model and experimental results. (DOC)

S1 Dataset Data set used in the manuscript. (ZIP)
