# Supplementary material for: Recommendation system in social networks with topical attention and probabilistic matrix factorization
Source: PLoS One. 2019 Oct 31;14(10):e0223967. doi: 10.1371/journal.pone.0223967 (PMC6822766; doi:10.1371/journal.pone.0223967)
Supplement: S1 File — (DOC) [file pone.0223967.s001.doc]

Table 1. Data statistics on two real-world datasets

| Datasets | #users | #items | #ratings | #trust |
| --- | --- | --- | --- | --- |
| Epinions | 22166 | 296277 | 922267 | 355754 |
| Ciao | 7375 | 106797 | 284086 | 111781 |

**Table 2. RMSE comparisons for different K**

|  | K=5 | | K=10 | | K=20 | |
| --- | --- | --- | --- | --- | --- | --- |
| Algorithm | Epinions | Ciao | Epinions | Ciao | Epinions | Ciao |
| PMF | 1.1347 | 1.0483 | 1.1491 | 1.0438 | 1.1581 | 1.0455 |
| SoRec | 1.0758 | 1.0308 | 1.1047 | 1.0645 | 1.2260 | 1.1620 |
| SoReg | 1.0674 | 0.9924 | 1.1109 | 1.0011 | 1.2399 | 1.0970 |
| RSTE | 1.0915 | 1.0471 | 1.1387 | 1.0300 | 1.2291 | 1.1656 |
| SocialMF | 1.0809 | 1.0087 | 1.0904 | 1.0700 | 1.1932 | 1.0060 |
| TrustMF | 1.0722 | 0.9794 | 1.1208 | 0.9823 | 1.2893 | 0.9812 |
| CTR | 1.0644 | 0.9543 | 1.0576 | 0.9662 | 1.0916 | 0.9678 |
| SDAE | 1.0457 | 0.9321 | 1.0501 | 0.9525 | 1.0732 | 0.9603 |
| STAPMF | 1.0301 | 0.9131 | 1.0416 | 0.9341 | 1.0478 | 0.9517 |


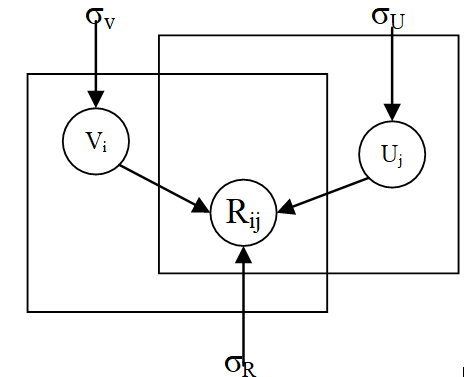


Fig 1. Probabilistic matrix factorization diagram model


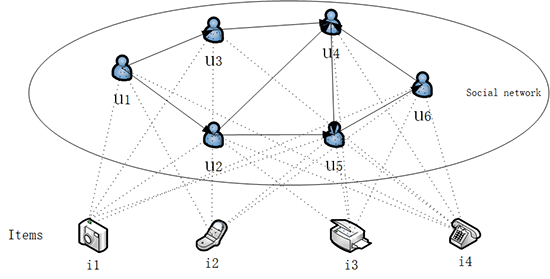

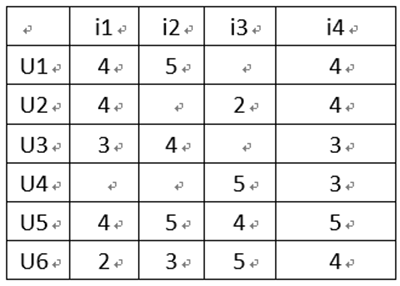


(a) User-item diagram representation (b) User-item-rating matrix

Fig 2. The expression of trust social network recommendations


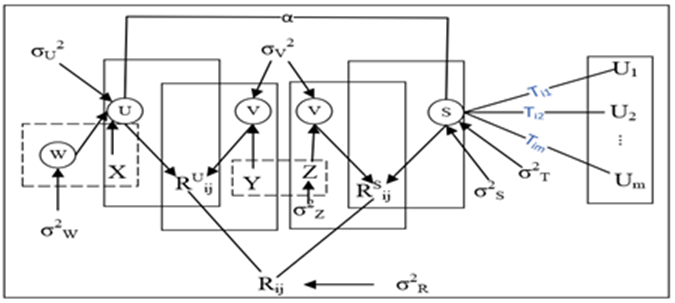


Figure 3. STAPMF model


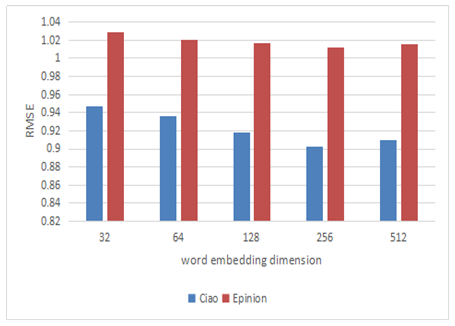

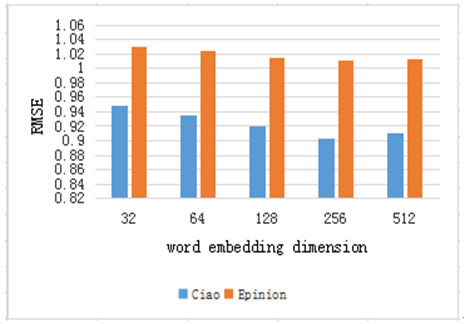


Fig 4. Validation RMSE as a result of varying $\text{d}_{W}$**,**$d_{Z}$

**The experimental results of Fig 4**

|  | Ciao | Epinion |
| --- | --- | --- |
| 32 | 0.9487 | 1.0302 |
| 64 | 0.9356 | 1.0245 |
| 128 | 0.9201 | 1.0141 |
| 256 | 0.9034 | 1.0105 |
| 512 | 0.9098 | 1.0131 |

|  | Ciao | Epinion |
| --- | --- | --- |
| 32 | 0.9472 | 1.0291 |
| 64 | 0.9364 | 1.0204 |
| 128 | 0.9175 | 1.0162 |
| 256 | 0.9027 | 1.0116 |
| 512 | 0.91015 | 1.0151 |


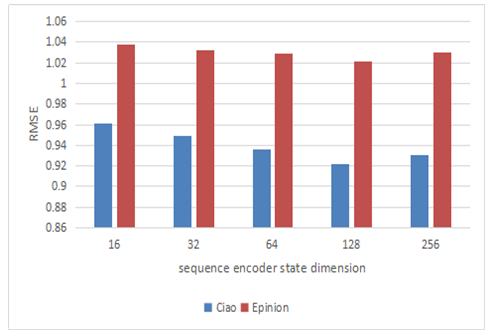

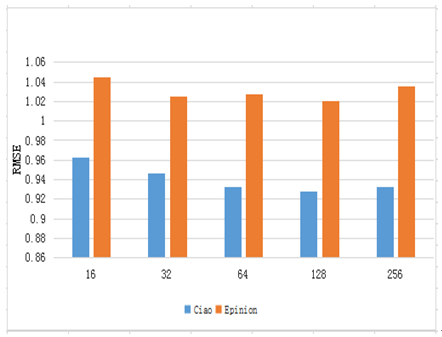


**Fig 5. Validation RMSE as a result of varying $d_{X}$,**$d_{Y}$

The experimental results of Fig 5

|  | Ciao | Epinion |
| --- | --- | --- |
| 16 | 0.9625 | 1.0453 |
| 32 | 0.9467 | 1.0251 |
| 64 | 0.9326 | 1.0276 |
| 128 | 0.9276 | 1.0201 |
| 256 | 0.9324 | 1.0352 |

|  | Ciao | Epinion |
| --- | --- | --- |
| 16 | 0.9615 | 1.0381 |
| 32 | 0.9486 | 1.0319 |
| 64 | 0.9365 | 1.0285 |
| 128 | 0.9218 | 1.0217 |
| 256 | 0.9304 | 1.0302 |


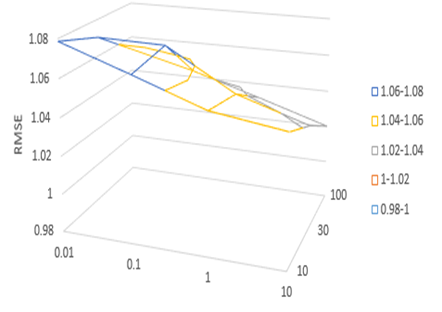

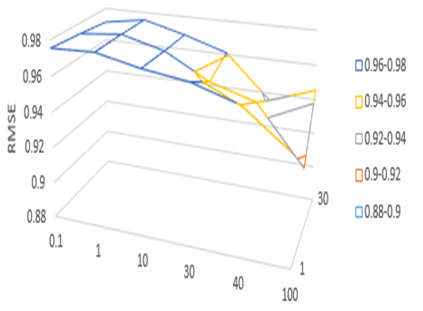


**Fig 6. Validation RMSE as a result of varying $\lambda_{u}$,**$\lambda_{v}$

The experimental results of Fig 6

|  | 1 | 10 | 30 |
| --- | --- | --- | --- |
| 0.1 | 0.9754 | 0.9737 | 0.9714 |
| 1 | 0.9763 | 0.9761 | 0.9754 |
| 10 | 0.9709 | 0.9695 | 0.9689 |
| 30 | 0.9674 | 0.9554 | 0.9609 |
| 40 | 0.9594 | 0.9477 | 0.9357 |
| 100 | 0.9391 | 0.9154 | 0.9454 |

|  | 10 | 30 | 100 |
| --- | --- | --- | --- |
| 0.01 | 1.0786 | 1.0702 | 1.0527 |
| 0.1 | 1.0659 | 1.0693 | 1.0434 |
| 1 | 1.0527 | 1.0474 | 1.0321 |
| 10 | 1.0474 | 1.0354 | 1.0203 |


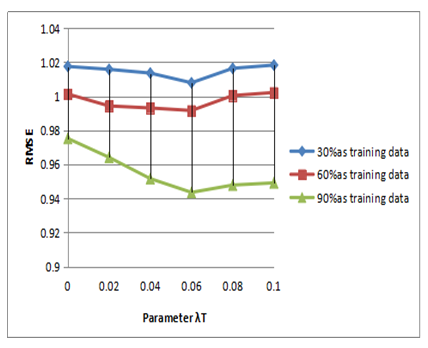

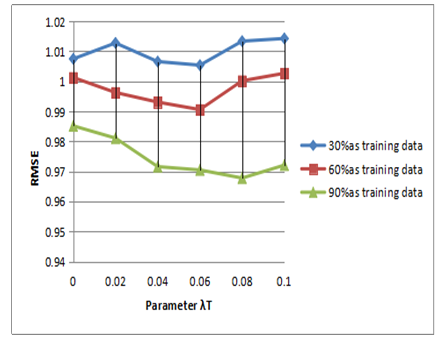


Fig 7. Parameter analysis of Ciao

The experimental results of Fig 7

|  | 30%as training data | 60%as training data | 90%as training data |
| --- | --- | --- | --- |
| 0 | 1.0175 | 1.0013 | 0.9752 |
| 0.02 | 1.0158 | 0.9943 | 0.9641 |
| 0.04 | 1.0136 | 0.9931 | 0.9516 |
| 0.06 | 1.0079 | 0.9916 | 0.9435 |
| 0.08 | 1.0164 | 1.0004 | 0.9478 |
| 0.1 | 1.0183 | 1.0022 | 0.9491 |

|  | 30%as training data | 60%as training data | 90%as training data |
| --- | --- | --- | --- |
| 0 | 1.0075 | 1.0013 | 0.9852 |
| 0.02 | 1.0128 | 0.9963 | 0.9811 |
| 0.04 | 1.0066 | 0.9931 | 0.9716 |
| 0.06 | 1.0054 | 0.9906 | 0.9705 |
| 0.08 | 1.0134 | 1.0002 | 0.9678 |
| 0.1 | 1.0143 | 1.0027 | 0.9721 |


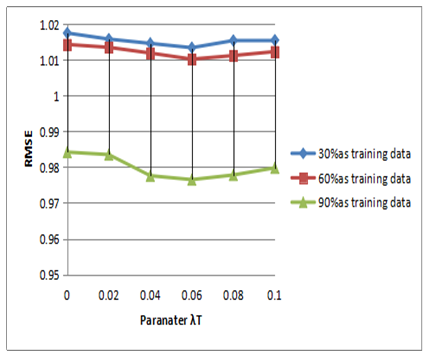

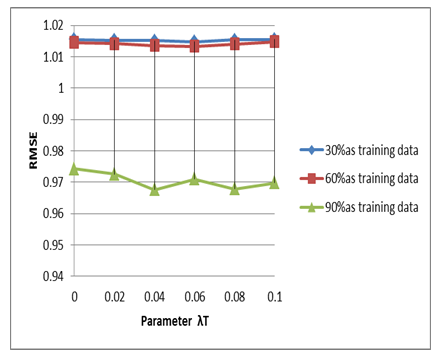


Fig 8. Parameter analysis of Epinions

The experimental results of Fig 8

|  | 30%as training data | 60%as training data | 90%as training data |
| --- | --- | --- | --- |
| 0 | 1.0175 | 1.0143 | 0.9842 |
| 0.02 | 1.0158 | 1.0135 | 0.9835 |
| 0.04 | 1.0146 | 1.0119 | 0.9776 |
| 0.06 | 1.0134 | 1.0102 | 0.9765 |
| 0.08 | 1.0154 | 1.0112 | 0.9778 |
| 0.1 | 1.0155 | 1.0123 | 0.9798 |

|  | 30%as training data | 60%as training data | 90%as training data |
| --- | --- | --- | --- |
| 0 | 1.0155 | 1.0145 | 0.9742 |
| 0.02 | 1.0153 | 1.0142 | 0.9725 |
| 0.04 | 1.0151 | 1.0136 | 0.9676 |
| 0.06 | 1.0148 | 1.0133 | 0.9709 |
| 0.08 | 1.0154 | 1.0141 | 0.9678 |
| 0.1 | 1.0155 | 1.0148 | 0.9698 |


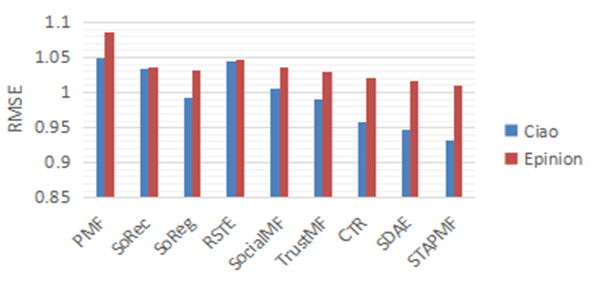


Fig 9. Comparison results on the different datasets.

The experimental results of Fig 9

|  | Ciao | Epinion |
| --- | --- | --- |
| PMF | 1.0479 | 1.0864 |
| SoRec | 1.0327 | 1.0365 |
| SoReg | 0.9931 | 1.0323 |
| RSTE | 1.0448 | 1.0466 |
| SocialMF | 1.0046 | 1.0352 |
| TrustMF | 0.9895 | 1.0291 |
| CTR | 0.9587 | 1.0204 |
| SDAE | 0.9467 | 1.0159 |
| STAPMF | 0.9316 | 1.0104 |
